# Impact of prior levonorgestrel intrauterine device use at the time of embryo transfer

**DOI:** 10.1530/RAF-24-0099

**Published:** 2024-12-20

**Authors:** Anna Vanderhoff, Andrea Lanes, Elizabeth Ginsburg

**Affiliations:** ^1^ Department of Obstetrics and Gynecology, Brigham and Women’s Hospital, Boston, Massachusetts 02130, USA; ^2^Harvard Medical School, Boston, Massachusetts 02115, USA

**Keywords:** levonorgestrel, intrauterine device, endometrial thickness, embryo transfer

## Abstract

**Abstract:**

Our objective is to investigate whether infertile women with a history of levonorgestrel intrauterine device (LNG IUD) use have impaired endometrial growth and pregnancy rates after embryo transfer. This is a retrospective cohort study at a single academic medical center of infertile women aged 18–44 with a history of LNG IUD use undergoing their first embryo transfer cycle between January 2019 and January 2021 compared to controls with a history of no prior birth control use (NONE) or prior oral contraceptive (OCP) use. The primary outcome is endometrial thickness prior to embryo transfer. Secondary outcomes are embryo transfer results, including implantation, miscarriage and live birth. Demographic, baseline and cycle characteristics were similar among the three groups. Women with a history of LNG IUD use had thinner endometrial stripes than women with a history of NONE (LNG IUD: 8.93 mm, NONE: 10.32 mm (aRR: 0.88, 95% CI: 0.80–0.97)) but not when compared to women with a history of OCP use (OCP: 9.61 mm (aRR: 0.92, 95% CI: 0.84–1.01)). Women with a history of LNG IUD use had slightly higher implantation rates than those with NONE history (LNG IUD: 43.37%, NONE: 24.17% (relative risk (RR): 1.79, 95% CI: 1.21–2.45)), though not when compared to prior OCP users (OCP: 38.72% (RR: 1.12, 95% CI: 0.86–1.47)). The remainder of the embryo transfer outcomes were similar among the three groups. In conclusion, prior LNG IUD users have a reduced endometrial thickness at the time of embryo transfer but do not have worse pregnancy outcomes.

**Lay summary:**

LNG IUDs (brand names include Mirena, Liletta and Skyla) are some of the most widely used forms of birth control. While these devices are safe and are generally not believed to impact fertility, a few recent articles have suggested the possibility of a long-term impact of prior intrauterine device use on the uterine lining in some patients. As such, we sought to examine uterine lining growth and outcomes of assisted reproduction in an infertile patient population with a history of LNG IUD use compared to infertile women without a history of LNG IUD use. Ultimately, we found that women with a history of LNG IUD use had less uterine lining growth during assisted reproduction cycles, but that they did not have worse pregnancy outcomes when compared to women without a history of levonorgestrel device use.

## Introduction

The levonorgestrel intrauterine device (LNG IUD) has been available as a birth control method in the United States since 2000 (ACOG Practice Bulletin 186 2017). IUD use has grown over the past 20 years, especially among young women (Kavanaugh & Jerman 2018). IUDs are now the most widely used contraceptive method in the world (The ESHRE Capri Workshop Group 2008). By 2017, more than 15% of women aged 25–34 in the United States reported that they had used an IUD for contraception within the prior 30 days (KFF Intrauterine Devices 2020).

One of the key mechanisms of action of the LNG IUD is localized administration of high concentrations of LNG, ranging from 470 to 1500 ng/g (Nilsson *et al.* 1986), which results in endometrial glandular atrophy, stromal decidualization, localized inflammation, and necrosis (Silverberg *et al.* 1986, Ortiz & Croxatto 2007, Dinh *et al.* 2015, Dinehart *et al.* 2020). This is facilitated, in part, by the down-regulation of endometrial estrogen and progesterone receptors and changes in endometrial gene expression (Jones & Critchley 2000, Rutanen 2000, Horcajadas *et al.* 2006, Engemise *et al.* 2011, Dinehart *et al.* 2020). While these effects are generally believed to reverse after LNG IUD removal (Silverberg *et al.* 1986, Horcajadas *et al.* 2006), there is evidence that there may be prolonged alteration of endometrial gene expression after IUD use in some women (Horcajadas *et al.* 2006, Dinehart *et al.* 2020). Furthermore, one case series demonstrated a possible association between Asherman’s syndrome and LNG IUD use (Abel 2021).

A substantial amount of literature has demonstrated high rates of conception after LNG IUD removal (Andolsek *et al.* 1986, Andersson *et al.* 1992, Skjeldestad 2008, Gemzell-Danielsson *et al.* 2017, Yland *et al.* 2020). However, a few of these studies have shown 12-month conception rates in the 70s to low 80s for women in their 20s and 30s (Andersson *et al.* 1992, Gemzell-Danielsson *et al.* 2017), which is slightly lower than the accepted population-level 12-month conception rates in the low 90s for similarly aged women (Gnoth 2003).

Taken together, findings of possible alteration in endometrial function and a reduction or delay in fecundity after LNG IUD use suggest an urgent need for further investigation into the possible impact of LNG IUDs on the outcome of assisted reproduction treatment in women with infertility and a history of LNG IUD use. As such, the goal of this study is to examine endometrial stripe thickness and embryo transfer outcomes for women who have previously used LNG IUDs as compared to women with no prior birth control use (NONE) and prior oral contraceptive (OCP) use.

## Materials and methods

This is a retrospective cohort study conducted at a single academic medical center. We examined first fresh or frozen embryo transfer cycle characteristics and pregnancy outcomes in patients with a history of LNG IUD use as compared to both patients with a history of NONE and patients with a history of prior OCP use. Only first embryo transfer cycles were included due to the potential for a mitigated impact of prior LNG IUD use in the setting of multiple hormonally stimulated cycles. Prior contraception usage was self-reported either on new patient questionnaires or at the initial patient visit at the Brigham and Women’s Hospital Center for Infertility and Reproductive Surgery. These data were complete for 82.1% of patients. We included women aged 18–44 undergoing their first embryo transfer cycle (fresh or frozen) between January 2019 and January 2021.

Potential participants were excluded if they utilized third-party reproduction via a donor egg or the use of a gestational carrier. They were also excluded if they utilized natural cycle, modified natural cycle or gonadotropin preparations for frozen embryo transfer cycles, as we do not routinely perform endometrial thickness checks during those cycles. Women with a history of other factors that could contribute to thin endometrial stripes and poor embryo transfer outcomes, including uterine factor infertility, history of pelvic radiation, or history of Asherman’s syndrome, were also excluded.

We also sent a survey to all participants regarding the length of prior LNG IUD use and timing of LNG IUD removal but had a very low response rate of 7% precluding useful analysis and inclusion in this report.

### Stimulated/fresh embryo transfer

A variety of assisted reproduction ovulation induction protocols were utilized. These were grouped into ‘good responder’ protocols, which included antagonist, pseudoluteal and low-dose luteal down-regulation, and ‘poor responder’ protocols, which included very low-dose luteal, ultra-low-dose luteal, microflare, patch and minimal stimulation. Some patients received up to 7 days of OCP lead-in prior to both fresh and frozen cycles. Given the lack of prolonged exposure, those patients were not reclassified into the prior OCP use group. Patients were monitored per standard protocols with laboratory testing for estradiol and progesterone as well as transvaginal ultrasounds during their cycles. In fresh transfer cycles, they received a trigger shot with either human chorionic gonadotropin alone or leuprolide and human chorionic gonadotropin when transvaginal ultrasound monitoring demonstrated at least two mean follicular diameters >18 mm. Endometrial stripe thickness for fresh embryo transfer cycles was considered the endometrial stripe thickness on their last monitoring ultrasound prior to retrieval and transfer. All monitoring ultrasounds were completed by experienced ultrasound technicians within the Brigham and Women’s Radiology Department using General Electric ultrasounds. We do not utilize endometrial stripe thickness to determine the appropriateness of embryo transfer in fresh cycles. Oocyte retrievals took place 36 h after trigger via transvaginal ultrasound guidance with monitored anesthesia care. Insemination took place via either traditional *in vitro* fertilization or intracytoplasmic sperm injection (ICSI) as clinically indicated. All embryos were cultured in standard one-step culture media. Fresh embryo transfers took place with ultrasound guidance at either the cleavage stage (3 days after retrieval) or blastocyst stage (5 days after retrieval). Embryo grading criteria are reported in Supplemental Tables 1 and 2 (see section on Supplementary materials given at the end of the article).

### Cryopreserved embryo transfer

We also examined outcomes after first frozen (cryopreserved) embryo transfer. Participants underwent medicated embryo transfer cycles. They had hormone studies completed at the beginning of the follicular phase to confirm menstrual timing and then started either oral, vaginal or transdermal estradiol supplementation according to provider and patient preference. Cycles that utilized leuprolide prior to progesterone start were not included. Progesterone supplementation was started after ultrasound confirmed >7 mm endometrial thickness and progesterone level confirmed no prior ovulation during the estrogen preparation phase. The first endometrial stripe check ultrasound typically takes place about 10 days after estradiol level conformed to be above 200 pg/mL. If a thin stripe was confirmed, estrogen doses were increased or the route of administration was changed, followed by a repeat ultrasound. The majority of patients undergoing cryopreserved embryo transfer who never reached an endometrial stripe thickness of 7 mm did not have a cryopreserved embryo transfer (CET) and were excluded from this study. A few patients did have transfers at less than 7 mm when directed by their providers, though none at less than 6 mm. Endometrial thickness in medicated cryopreserved embryo transfer cycles was considered the final endometrial thickness measurement prior to progesterone start. Progesterone was administered either intramuscularly or vaginally. Embryo transfers then took place via ultrasound guidance after either three nights of progesterone supplementation (cleavage stage) or five nights of progesterone supplementation (blastocyst stage).

### Outcomes

The primary outcome was pre-transfer endometrial thickness. Secondary outcomes included implantation rate, defined as the number of gestational sacs on the first obstetric ultrasound per number of transferred embryos; chemical pregnancy rate, defined as positive human chorionic gonadotropin without sonographic evidence of a pregnancy; ectopic pregnancy rate, defined as the presence of a pregnancy outside of the uterus; miscarriage rate, defined as the loss of a previously seen intrauterine pregnancy prior to 24 weeks; transferred to OB care, defined as an ongoing pregnancy beyond 8 weeks; and live birth rate, defined as a live birth beyond 24 weeks.

### Statistical analysis

Means and standard deviations were generated for continuous variables, and frequencies and proportions for categorical variables. Chi-square tests and Fisher’s exact tests were performed for categorical variables where appropriate. Kruskal–Wallis tests were performed for continuous variables. Relative risks (RRs) and 95% confidence intervals were generated using log-binomial regression for categorical outcomes, Poisson regression for counts and Poisson regression with an offset for rates. Models assessing endometrial thickness were adjusted for age, BMI and fresh vs frozen transfer. Models assessing embryo transfer outcomes were adjusted for age, BMI, embryo quality, number of embryos transferred, use of pre-implantation genetic testing for aneuploidy, day of transfer and fresh vs frozen embryo transfer. Adjusted RRs were not included when the model did not converge. An alpha of 0.05 was considered statistically significant. All appropriate tests were two-sided. All statistical analyses were performed with SAS, version 9.4 (USA; https://www.sas.com/).

### Ethical approval

Approval for this study and exemption from informed consent was obtained from the Massachusetts General Brigham Institutional Review Board (protocol number 2022P002017).

## Results

We analyzed data from a total of 616 patients who had their first fresh or frozen embryo transfer at Brigham and Women’s Hospital between January 2019 and January 2021. Of those women, 197 reported NONE history, 357 reported only prior OCP use, and 62 reported prior LNG IUD use and were included in the analysis. Table 1 represents participants’ baseline characteristics. The mean age was 36.62 for women with NONE, 35.28 for women with prior OCP use and 34.71 for women with a history of LNG IUD use (*P* < 0.01). Mean BMI (kg/m^2^) was around 26 for all three groups (NONE 26.35, OCP 26.74, LNG IUD 26.00). Women with a history of NONE were more likely to be Asian (NONE 35.71%, OCP 10.51%, LNG IUD 4.84%) and Black (NONE 12.76%, OCP 2.84% LNG IUD 6.45%) than women with a history of OCP use and women with a history of LNG IUD use (*P* < 0.01).

**Table 1:** Baseline characteristics of patients categorized by contraceptive use. Data are presented as mean ± SD or as *n* (%).

Characteristics	NONE (*n* = 197)	OCP (*n* = 357)	LNG IUD (*n* = 62)	*P* value
Age (years)				
Mean ± SD	36.62 ± 4.15	35.28 ± 3.97	34.71 ± 3.91	<0.01
Min–max	27.10–44.80	24.50–44.70	27.30–43.80	
BMI (kg/m^2^)				
Mean ± SD	26.35 ± 6.40	26.74 ± 7.14	26.00 ± 7.03	0.66
Min–max	17.57–51.82	17.57–54.75	17.74–49.08	
Race, *n* (%)				
Asian	70 (35.71)	37 (10.51)	3 (4.84)	<0.01
Black	25 (12.76)	10 (2.84)	4 (6.45)	
Caucasian	84 (42.86)	294 (83.52)	54 (87.10)	
Other^*^	17 (8.67)	11 (3.13)	1 (1.61)	
Missing	1	5	0	
Infertility diagnosis, *n* (%)				
DOR	30 (15.23)	52 (14.57)	8 (12.90)	<0.01
Ovulatory dysfunction	3 (1.52)	33 (9.24)	3 (4.84)	
Tubal factor	14 (7.11)	9 (2.52)	3 (4.84)	
Endometriosis	3 (1.52)	9 (2.52)	2 (3.23)	
Male factor	28 (14.21)	53 (14.85)	13 (20.97)	
Other	22 (11.17)	45 (12.61)	17 (27.42)	
Unexplained	62 (31.47)	93 (26.05)	4 (6.45)	
Male + female factor	22 (11.17)	35 (9.80)	10 (16.13)	
Multiple female factors	13 (6.60)	28 (7.84)	2 (3.23)	
AMH (ng/mL)				
Mean ± SD	2.83 ± 2.72	3.26 ± 3.08	2.54 ± 1.88	0.31
Min–max	0.08–17.46	0.04–21.35	0.10–8.6)	
Missing	1	0	0	
Day 3 FSH (pg/mL)				
Mean ± SD	8.71 ± 6.07	8.59 ± 6.82	8.47 ± 3.87	0.41
Min–max	0.80–62.00	0.10–85.80	2.40–25.10	
Missing	3	9	2	
History of PID, *n* (%)				
No	186 (94.42)	343 (96.08)	62 (100.00)	0.13
Yes	11 (5.58)	14 (3.92)	0 (0.00)	
History of uterine surgery, *n* (%)				
No	147 (74.62)	304 (85.15)	47 (75.81)	<0.01
Yes	50 (25.38)	53 (14.85)	15 (24.19)	

AMH, anti-Mullerian hormone; FSH, follicle-stimulating hormone; LNG IUD, levonorgestrel intrauterine device; NONE, no prior birth control; OCP, oral contraceptive.

* indicates Native Hawaiian or Pacific Islander, American Indian or Alaska Native.

Rates of different primary infertility diagnoses were relatively similar across the three groups, with a few exceptions. Women with a history of only prior OCP use were slightly more likely to have a primary infertility diagnosis of ovulatory dysfunction (NONE: 1.52%, OCP: 9.24%, LNG IUD: 4.84%). Participants with prior LNG IUD use were more likely than the other two groups to have other as their primary infertility diagnosis (NONE: 11.17%, OCP: 12.61%, LNG IUD: 27.42%) and less likely to have unexplained infertility as their primary infertility diagnosis (NONE: 31.47%, OCP: 26.05%, LNG IUD: 6.45%). Women with a history of LNG IUD use were also slightly more likely to have a primary infertility diagnosis of male factor infertility (NONE: 14.21%, OCP: 14.85%, LNG IUD: 20.97%). Baseline ovarian reserve testing parameters, including anti-Mullerian hormone (NONE: 2.83 ng/mL, OCP: 3.26 nL/mL, LNG IUD: 2.54 ng/mL) and cycle day three follicle-stimulating hormone levels, were similar across all groups. A small number of patients had a history of pelvic inflammatory disease in all three groups (NONE: 5.58%, OCP: 3.92%, LNG IUD: 0.00%). Slightly fewer patients in the OCP group had a history of any uterine surgery when compared to women with a history of NONE and women with a history of prior LNG IUD use (NONE: 25.38%, OCP: 14.85%, LNG IUD: 24.19%, *P* < 0.01).

Table 2 reports the characteristics of the fresh stimulation cycles from which the embryos transferred in first fresh or cryopreserved embryo transfer cycles resulted, as well as the characteristics of those embryo transfer cycles. Women with a history of NONE were slightly more likely to be prescribed poor responder fresh stimulation protocols (NONE: 19.29%, OCP: 14.75%, LNG IUD: 14.52%). All three groups received similar total gonadotropin doses (NONE: 3947.21 IU, OCP: 3926.39 IU, LNG IUD: 4034.27 IU), had clinically equivalent lengths of stimulation (NONE: 11.77 days, OCP: 12.13 days, LNG IUD: 11.84 days) and had comparable maximum estradiol levels at the time of the trigger shot (NONE: 2274.95 pg/mL, OCP: 2136.78 pg/mL, LNG IUD: 2057.62 pg/mL). Women with a history of LNG IUD use were slightly more likely to utilize ICSI for oocyte fertilization (NONE: 44.16%, OCP: 47.62%, LNG IUD: 58.06%), likely due to higher rates of male factor infertility in this group.

**Table 2:** Fresh and cryopreserved cycle characteristics by IUD use. Data are presented as mean ± SD or as *n* (%).

Characteristics	NONE	OCP	LNG IUD	*P* value
Fresh stimulation cycle				
*n*	197	357	62	
Cycle type, *n* (%)				
Poor responder^*^	38 (19.29)	52 (14.57)	9 (14.52)	0.28
Good responder^†^	159 (80.71)	302 (84.59)	52 (83.87)	
OI to IVF conversion	0 (0.00)	3 (0.84)	1 (1.61)	
Total gonadotropin dose (IU)				
Mean ± SD	3947.21 ± 2062.67	3926.39 ± 2149.39	4034.27 ± 2115.14	0.92
Min–max	1050–11,775	825–13,500	975–9600	
Estradiol level at trigger (pg/mL)				
Mean ± SD	2274.95 ± 1218.20	2136.78 ± 1102.82	2057.62 ± 1029.62	0.24
Min–max	606–9983	265–6519	237–5251	
Day of trigger				
Mean ± SD	11.77 ± 2.10	12.13 ± 2.67	11.84 ± 2.56	0.24
Min–max	8–21	8–32	9–22	
Trigger type, *n* (%)				
HCG	108 (54.82)	172 (48.18)	32 (51.61)	0.68
HCG/Lupron	58 (29.44)	121 (33.89)	19 (30.65)	
Lupron	31 (15.74)	64 (17.93)	11 (17.74)	
ICSI, *n* (%)				
No	110 (55.84)	187 (52.38)	26 (41.94)	0.16
Yes	87 (44.16)	170 (47.62)	36 (58.06)	
Number of mature eggs				
Mean ± SD	9.85 ± 7.76	11.11 ± 8.64	10.66 ± 6.55	
Min–max	1–46	1–50	1–31	
RR (95% CI)^‡^	NA	1.13 (1.07–1.19)^††^	1.08 (0.99–1.18)	
aRR (95% CI)^‡^^,^^§^	NA	0.95 (0.87–1.05)	0.94 (0.86–1.02)	
Number of 2PNs^║^				
Mean ± SD	8.72 ± 6.69	9.89 ± 7.87	9.40 ± 5.85	
Min–max	1–35	1–44	1–30	
RR (95% CI)^‡^	NA	1.13 (1.07–1.20)^††^	1.08 (0.98–1.18)	
aRR (95% CI)^‡^^,^^§^	NA	0.98 (0.89–1.09)	0.97 (0.88–1.06)	
Fertilization rate				
Mean ± SD	90.73 ± 16.41	89.49 ± 17.89	90.88 ± 14.68	
Min–max	17.39–100.00	8.33–100.00	26.92–100.00	
RR (95% CI)^‡^	NA	1.01 (0.96–1.07)	1.06 (0.97–1.17)	
aRR (95% CI)^‡^^,^^§^	NA	1.05 (0.95–1.16)	1.05 (0.96–1.15)	
Fresh embryo transfer cycle				
*n*	146	230	43	
Route of PGR supplementation, *n* (%)				
Intramuscular	1 (0.68)	2 (0.87)	0 (0.00)	0.32
Vaginal	145 (99.32)	228 (99.13)	43 (100.00)	
Programmed cryopreserved embryo transfer cycle				
*n*	51	127	19	
Type of estrogen supplementation, *n* (%)				
Oral	40 (78.43)	95 (74.80)	11 (57.89)	0.46
Vaginal	3 (5.88)	8 (6.30)	2 (10.53)	
Transdermal	0 (0.00)	2 (1.57)	1 (5.26)	
Multiple types	8 (15.69)	22 (17.32)	5 (26.32)	
Type of PGR supplementation, *n* (%)				
Intramuscular	42 (82.35)	113 (88.98)	17 (89.47)	0.48
Vaginal	9 (17.65)	14 (11.02)	2 (10.53)	
Length of prep (days)^¶^				
Mean ± SD	15.45 ± 2.03	17.482 ± 4.45	17.32 ± 4.5	0.03
Min–max	14–22	11–33	11–32	
PGT-A embryos, *n* (%)				
Yes	20 (39.22)	59 (46.46)	12 (63.16)	0.20
No	31 (60.78)	68 (53.54)	7 (36.84)	
Fresh and cryopreserved embryo transfer				
*n*	197	357	62	
Embryo transfer stage, *n* (%)				
Cleavage	92 (46.70)	128 (35.85)	17 (27.42)	0.01
Blastocyst	105 (53.30)	229 (64.15)	45 (72.58)	
Embryo quality^**^, *n* (%)				
Excellent	56 (28.43)	83 (23.25)	16 (25.81)	0.68
Good	85 (43.15)	153 (42.86)	28 (45.16)	
Fair	36 (18.27)	85 (23.81)	14 (22.58)	
Poor	20 (10.15)	36 (10.08)	4 (6.45)	
Number of embryos transferred				
Mean ± SD	1.68 ± 1.23	1.40 ± 0.91	1.34 ± 0.85	0.01
Min–max	1–7	1–7	1–5	

2PNs, two pronuclear embryos; AMH, anti-Mullerian hormone; ICSI, intracytoplasmic sperm injection; IVF, *in vitro* fertilization; LNG IUD, levonorgestrel intrauterine device; NONE, no prior birth control; OCP, oral contraceptive; PGR, progesterone; RR, relative risk.

* very low dose luteal, ultra-low dose luteal, microflare, minimal stimulation, patch.

† antagonist, low-dose luteal, pseudoluteal.

^‡^ No birth control is the reference group.

^§^ Adjusted for age, BMI, race, diagnosis, AMH, total gonadotropin dose.

║ appropriately fertilized day one embryos with two pronuclei.

¶ number of days from cycle day one to day of mapping.

** See Supplemental Tables 1 and 2 for additional information on embryo quality and grading.

^††^ Significant value.

There were no significant differences in mature oocyte yield among the three groups (OCP: 11.11, NONE: 9.85, LNG IUD: 10.66). All three groups had similar fertilization rates at around 90% (NONE: 90.73%, OCP: 89.49%, LNG IUD: 90.88%). Women with a history of OCP use similarly had a slightly higher number of two pronuclear embryos (2PNs) than women with a history of NONE (OCP: 9.89, NONE: 8.72 (RR: 1.13, 95% CI: 1.07–1.20)), but that difference did not hold after adjustment (aRR: 1.05, 95% CI: 0.99–1.12). Former LNG IUD users had a similar number of 2PNs when compared to women with a history of NONE (LNG IUD: 9.40, NONE: 8.72 (aRR: 1.04, 95% CI: 0.96–1.17)).

Women who underwent fresh transfers overwhelmingly utilized vaginal progesterone supplementation (NONE: 99.32%, OCP: 99.13%, LNG IUD: 100.00%; Table 2). Most women who completed programmed cryopreserved embryo transfer cycles underwent estrogen priming with oral estradiol (NONE: 78.43%, OCP: 74.80%, LNG IUD: 57.89%). Women with a history of LNG IUD use and those with a history of OCP use had slightly longer lengths of estrogen priming prior to progesterone start (NONE: 15.45 days, OCP: 17.482 days, LNG IUD: 17.32 days, *P =* 0.03). Across all three groups, most patients utilized intramuscular progesterone supplementation for programmed cryopreserved embryo transfer cycles (NONE: 82.35%, OCP: 88.98%, LNG IUD: 89.47%). More women in the LNG IUD group had transfers at the blastocyst stage (NONE: 53.30%, OCP: 64.15%, LNG IUD: 72.58%, *P =* 0.01), and likely as a result, they had a lower number of embryos transferred per cycle than women in the other two groups (NONE: 1.68, OCP: 1.40, LNG IUD: 1.34, *P =* 0.01). Embryo quality at the cleavage and blastocyst stage was equivalent across all three groups.

Table 3 demonstrates endometrial thickness prior to embryo transfer and embryo transfer outcomes. Women with a history of LNG IUD use had slightly thinner endometrial stripes prior to embryo transfer than women with a history of NONE (LNG IUD: 8.93 mm, NONE: 10.32 mm (aRR: 0.88, 95% CI: 0.80–0.97)), but not when compared to women with a history of prior OCP use (LNG IUD: 8.93 mm, OCP: 9.61 mm (aRR: 0.92, 95% CI: 0.84–1.01)) when fresh and frozen cycles were considered together as an aggregate. This also held true when considering fresh and frozen cycles separately (fresh endometrial thickness mean: NONE = 10.53 mm, LNG IUD = 8.97 mm; frozen endometrial thickness mean: NONE = 9.69 mm, LNG IUD = 8.99 mm). The implantation rate was slightly higher for women with a history of LNG IUD use than for women with NONE (LNG IUD: 43.37%, NONE: 24.17% (RR: 1.78, 95% CI: 1.31–2.45)) and was similar to women with a history of OCP use (LNG IUD: 43.37%, OCP: 38.72% (RR: 1.12, 95% CI: 0.86–1.47)). Participants across all three groups had similar rates of chemical pregnancies (NONE: 13.71%, OCP: 10.08%, LNG IUD: 14.52%), ectopic pregnancies (NONE: 1.02%, OCP: 1.68%, LNG IUD: 0.00%) and spontaneous abortions (NONE: 9.14%, OCP: 7.84%, LNG IUD: 6.45%). Women with a history of LNG IUD use appeared to have a slightly higher rate of ongoing pregnancy beyond 10 weeks (LNG IUD: 48.39%, NONE: 30.96% (RR: 1.56, 95% CI: 1.12–2.18)) and live birth (LNG IUD: 46.77%, NONE: 30.46% (RR: 1.54, 95% CI: 1.09–2.16)) when compared to women without a history of prior birth control use, although those differences did not hold after adjustment. There were no statistically significant differences in rates of ongoing pregnancy beyond 8 weeks or live birth between women with a history of LNG IUD use and women with a history of OCP use.

**Table 3:** Embryo transfer outcomes by contraceptive use.

Outcomes	NONE (*n* = 197)	OCP (*n* = 357)	LNG IUD (*n* = 62)	LNG IUD vs NONE^*^	LNG IUD vs OCP^*^
Endometrial thickness					
Mean ± SD	10.32 ± 2.64	9.61 ± 2.53	8.98 ± 3.03	0.87 (0.79–0.96)^§^	0.93 (0.86–1.02)
Min–max	3.20–19.80	4.00–18.70	4.30–20.95	0.88 (0.80–0.97)*	0.92 (0.84–1.01)
Implantation rate^†^					
Sacs/transferred	80/331	194/501	36/83	1.79 (1.31–2.45)^§^	1.12 (0.86–1.47)
Rate	24.17	38.72	43.37	NA^‡^	NA^‡^
Chemical pregnancy					
No	170 (86.29)	321 (89.92)	53 (85.48)	1.06 (0.53–2.13)	1.44 (0.73–2.84)
Yes	27 (13.71)	36 (10.08)	9 (14.52)	NA^‡^	NA^‡^
Ectopic pregnancy					
No	195 (98.98)	351 (98.32)	62 (100.00)	NA^‡^	NA^‡^
Yes	2 (1.02)	6 (1.68)	0 (0.00)	NA^‡^	NA^‡^
Spontaneous abortion					
No	179 (90.86)	329 (92.16)	58 (93.55)	0.71 (0.25–2.01)	0.82 (0.30–2.26)
Yes	18 (9.14)	28 (7.84)	4 (6.45)	NA^‡^	NA^‡^
Transferred to obstetric care					
No	136 (69.04)	206 (57.70)	32 (51.61)	1.56 (1.12–2.18)^§^	1.14 (0.86–1.52)
Yes	61 (30.96)	151 (42.30)	30 (48.39)	1.24 (0.87–1.76)	0.99 (0.75–1.31)
Live birth					
No	137 (69.54)	209 (58.54)	33 (52.23)	1.54 (1.09–2.16)^§^	1.13 (0.84–1.51)
Yes	60 (30.46)	148 (41.46)	29 (46.77)	1.23 (0.86–1.78)	0.97 (0.73–1.30)

LNG IUD, levonorgestrel intrauterine device; NONE, no prior birth control; OCP, oral contraceptive; RR, relative risk.

* Adjusted for age, BMI, race, diagnosis, embryo quality, number of embryos transferred, transfer day, fresh/frozen transfer, use of PGT-A, endometrial thickness (except where that is the outcome). The values are RR (95% CI) or aRR (95% CI).

† number of gestational sacs/number of transferred embryos.

^‡^ Algorithm did not converge.

^§^ Significant value.

## Discussion

This study demonstrates that in an infertile patient population, there may be an impaired endometrial thickness in response to stimulation during embryo transfer cycles for women who have a history of LNG IUD use as compared to women without a history of birth control use. In contrast, women with a history of prior OCP use do not have an impaired endometrial stripe development when compared to women without a history of prior birth control use. While the actual difference in endometrial thickness was not large – 8.93 mm for LNG IUD users compared to 10.32 mm for women with no history of birth control use – it supports prior literature, which suggests that there may be some patients in whom there is a prolonged, if not permanent, alteration in endometrial function after LNG IUD or prolonged progesterone use (Horcajadas *et al.* 2006, Dinehart *et al.* 2020, Abel 2021). Reassuringly, despite thinner endometrial stripes, our study did not demonstrate impaired implantation and pregnancy rates after embryo transfer for patients with a history of LNG IUD use when compared to patients without a history of prior birth control use or those with a history of only prior OCP use. If anything, there may be a trend toward improved embryo transfer success for prior LNG IUD users. This finding does agree with prior literature, which suggests no meaningful fertility impact of LNG IUD use across a broad population (Andolsek *et al.* 1986, Andersson *et al.* 1992, Skjeldestad 2008, Gemzell-Danielsson *et al.* 2017, Yland *et al.* 2020). It also aligns with recent work by Ata and coworkers, which suggests that embryo transfer success may not be related to endometrial thickness (Ata *et al.* 2023). However, we should note that we may not capture the highest risk group – those with a prolonged history of LNG IUD use – adequately in this study and that the group deserves additional investigation. It is also important to point out that <10% of our patient population reported IUD usage, which is lower than the national norm of 15% (KFF Intrauterine Devices 2020). This may be because we have an older patient population than that quoted in the national IUD prevalence data.

The strengths of this study include that all patients had their retrievals and transfers at a single academic center with standard protocols, which helps limit confounding. In addition, to our knowledge, this is also the first investigation to date to examine the effects of the LNG IUD on endometrial stripe thickness in response to stimulation, and implantation and pregnancy rates after embryo transfer in an infertile patient population. The main limitations of this study are the retrospective nature, which introduces a lack of ability to control for important covariates, such as health literacy and access to care; small patient population; heterogeneous treatment plans; and lack of information regarding the length of prior LNG IUD or other birth control use and timing of LNG IUD removal in relation to embryo transfer. Of note, we did attempt to gather more detailed information on prior LNG IUD use than is present in the medical record via a survey that was sent to all patients in this study, but we had an extremely low response rate (7%) and thus were not able to include a rigorous analysis of those data in this report.

In conclusion, this study adds to the growing literature that suggests a potential for prolonged effects of the LNG IUD. This topic deserves expedited, in-depth future investigation so that we as a field can better understand the long-term impact of LNG IUD use and adequately counsel our patients on the fertility implications of LNG IUD use.

## Supplementary materials



## Declaration of interest

The authors declare that there is no conflict of interest that could be perceived as prejudicing the impartiality of this work.

## Funding

This work did not receive any specific grant from any funding agency in the public, commercial or not-for-profit sector.

## Author contribution statement

AV conceived the study, sought IRB approval, collected data, reviewed the results, and primarily authored this manuscript. AL conceived the study, analyzed the data, reviewed the results, and edited this manuscript. EG conceived the study, reviewed the results, edited this manuscript, and served as the main mentor for this project.
