# Supplementary material for: Impact of prior levonorgestrel intrauterine device use at the time of embryo transfer
Source: Reprod Fertil. 2024 Dec 20;5(4):e240099. doi: 10.1530/RAF-24-0099 (PMC11729790; doi:10.1530/RAF-24-0099)
Supplement: Supplementary file 1 [file supplementary_materials.pdf]

Supplementary Table 1: D5 Embryo quality grading

**Quality Group**	**Stage**	**ICM/TE**
Excellent	8 or 9	Aa, Ab, Ba, Bb, Ac, Bc, Ca, Cb
Good	7	Aa, Ab, Ba, Bb, Ac, Ca
	6 or 5	Aa
Fair	7	Bc, Cb
	6	Ab, Ba, Bb, Bc, Ac,
	5	Ba, Ab, Bb
Poor	6	Ca, Cb
	5	Ac, Bc, Ca, Cb
	4A or 4B	
Very poor	All others	

Supplementary Table 2: D3 Embryo quality grading

**Day3 Class**	**Cell# Frag Symm**
**1 (excellent)**	>801, >811, 801, 811, 802, 701
**2 (good)**	>802, >812, 821, 812, 711, 702,
	713, 601, 602
**3 (fair)**	822, 712, 722, 611, 621, 631, 612,
	622, 632, 623
**4 (poor)**	All others
